# Differences in the Association between Depression and Opioid Misuse in Chronic Low Back Pain *versus* Chronic Pain at Other Locations

**DOI:** 10.3390/healthcare4020034

**Published:** 2016-06-16

**Authors:** Arpana Jaiswal, Jeffrey F. Scherrer, Joanne Salas, Carissa van den Berk-Clark, Sheran Fernando, Christopher M. Herndon

**Affiliations:** 1 Department of Family and Community Medicine, Saint Louis University School of Medicine, St. Louis, MO 63104, USA; jaiswala@slu.edu (A.J.); salasj@slu.edu (J.S.); cvanden1@slu.edu (C.B.-C.); fernandos@slu.edu (S.F.); cherndo@siue.edu (C.M.H.); 2 Department of Pharmacy Practice, School of Pharmacy, Southern Illinois University Edwardsville, Edwardsville, IL 62026, USA

**Keywords:** chronic pain, pain location, depression, opioid misuse

## Abstract

Patients with chronic pain and depression are more likely to develop opioid abuse compared to patients without depression. It is not known if this association differs by pain location. We compared the strength of association between depression and opioid misuse in patients with chronic low back pain (CLBP) *vs.* chronic pain of other location (CPOL). Chart abstracted data was obtained from 166 patients seeking care in a family medicine clinic. Depression was measured by the PHQ-9 and opioid misuse was measured using the Current Opioid Misuse Measure. Pain severity and interference questions came from the Brief Pain Inventory. Cross-tabulations were computed to measure the association between depression and opioid misuse stratified on pain location. Exploratory logistic regression modeled the association between depression and opioid misuse after adjusting for pain location and pain severity and interference. Depression was significantly associated with opioid misuse in CPOL but not in CLBP. Regression results indicate pain interference partly accounts for the depression–opioid misuse association. These preliminary results from a small patient sample suggest depression may co-occur with opioid misuse more often in CPOL than in CLBP. Further research is needed to compare this comorbidity in specific pain diagnoses such as arthritis, fibromyalgia and CLBP.

## 1. Introduction

Patients with comorbid chronic pain and depression are more likely to receive a longer duration of opioid treatment at higher doses and are more likely to develop opioid abuse compared to non-depressed patients [1,2]. To our knowledge, it is not known if the link between depression and opioid abuse is similarly present in all types of chronic pain or is more common in chronic low back pain (CLPB). We are aware of one study of pain location and opioid misuse which found prevalence of opioid misuse was 82.8% in CLBP, 76.8% in those with arthritis, and 87.9% in neck/joint pain diagnosis suggesting some variation in opioid misuse by pain location [3]. The association between opioid misuse and depression could be stronger in CLBP given some evidence from patient populations that this pain location is associated with more depression than other pain types such as migraine and nerve root pain [4]. Comparison of fibromyalgia to other pain diagnostic groups found prevalence of the personality profile for neuroticism was greatest in fibromyalgia (46.2%) followed by 29.2% for lumbar, 20.5% for lower extremity and 15.4% for thoracic [5]. These results indicate that back pain patients have higher neuroticism compared to patients with other pain diagnoses with the exception of fibromyalgia. Significantly greater psychological distress and higher geriatric depression measures were reported for geriatric patients with CLBP compared to those with joint pain [6]. Data from survey studies indicate the prevalence and incidence of depression varies by pain location [7,8,9].

Thus, this limited literature provides some evidence that CLBP is associated with more depression and psychological distress than other pain types with the exception of fibromyalgia when data are obtained from patient populations. Given the strong association between opioid misuse and depression, we hypothesized that patients with CLBP would have a higher prevalence of depression and opioid misuse and this association would be stronger in CLBP than in patients with Chronic Pain of Other Location (CPOL).

We computed exploratory analysis to determine if: (1) the prevalence of depression and risk of opioid misuse differed among patients with CLBP *vs.* those with CPOL; and (2) the association between depression and risk of opioid misuse differed in patients with CLBP *vs.* those with CPOL.

## 2. Materials and Methods

### 2.1. Study Design and Population

Data for this cross-sectional study were obtained from a retrospective chart review of 166 chronic pain patients receiving pain management services at an academic family medicine clinic between January 2013 and December 2014. As part of routine care, patients presenting to the service completed the Brief Pain Inventory (BPI), Patient Health Questionnaire-9 (PHQ-9) and the Current Opioid Misuse Measure (COMM) at each visit. Data from initial clinical encounters were abstracted into an anonymized data set for the present study. After removing patients with missing data on any variable, the analytic data file contained 122 patients.

Pain conditions: From 22 primary pain diagnoses, we created a binary variable indicating the patient had CLBP, (n = 61) or CPOL (n = 61). Conditions among the CPOL group were osteoarthritis (n = 18), cervicalgia (n = 10), fibromyalgia (n = 7), migraine (n = 6), rheumatoid arthritis (n = 4), neuropathic pain (n = 4), HIV neuropathy (n = 2), avascular necrosis of the hip (n = 2), temporomandibular joint pain (n = 1), osteogenesis imperfecta (n = 1), lymphedema (n = 1), lower extremity trauma (n = 1), diffuse idiopathic skeletal hyperostosis (n = 1), complex regional pain syndrome (n = 1), chronic post stroke pain (n = 1) and abdominal pain (n = 1).

### 2.2. Measures

The three main measures assessed in this study were depression, risk of opioid misuse and pain interference.

#### 2.2.1. Depression

The PHQ-9 questionnaire was utilized to assess depression. PHQ-9 is a self-report tool used in screening, diagnosing, monitoring and measuring severity of depression. The tool measures Diagnostic and Statistical Manual of Mental Disorders—4th Edition (DSM-IV) depression symptoms with nine items. Scores range from 0–27 and suggested thresholds are 5 (mild), 10 (moderate), 15 (moderately severe) and 20 (severe depression) [10]. Due to sample size, we used a binary indicator of depression with a threshold of at least 15 for depression. We used this threshold to ensure patients had clinically relevant depression associated with seeking care and receiving treatment.

#### 2.2.2. Opioid Misuse

The Current Opioid Misuse Measure (COMM) questionnaire was used to measure risk of opioid misuse [11]. This questionnaire reflects patient’s risk of medication-related aberrant behavior among chronic pain patients using prescription opioids. The COMM includes 17-items ranging from 0–4. A summative score more than 9 is considered an elevated risk for opioid misuse. For ease of presentation, we refer to patients with this score as positive for risk of opioid misuse.

#### 2.2.3. Pain Severity and Interference

We used the Brief Pain Inventory (BPI) short form [12] as a tool for measuring pain severity and pain interference. Pain severity had four questions measuring least pain, worst pain, average pain and current pain score, with each item measured on a 0–10 Likert scale (10 being the worst). Pain interference contained seven questions measuring interference with: activity, mood, walking, work, relationships, sleep, and joy using the same 0–10 Likert scale. Following BPI scoring instructions, we created the average pain severity score by averaging all four pain scores (least pain, worst pain, average pain and current pain score) and similarly created an average pain interference score by averaging the seven interference components.

#### 2.2.4. Demographic Variables

Demographic data available from chart abstraction included age, gender and race. Due to small numbers of minority groups other than African-Americans (AA) we created a binary white/non-white race variable.

This study was approved by the IRBs of participating institutions.

### 2.3. Statistical Analysis

Frequencies, means and associations were computed using SPSS 23 [13]. Differences between CPOL and CLBP in demographics, pain indicators, depression, and opioid misuse risk were assessed using chi-square tests for categorical variables and independent samples t-tests for continuous variables. Also, chi-square and independent samples t-tests were conducted and stratified on pain location, to determine if there were differences in the association between depression and no depression with opioid misuse risk and pain. Similarly, the associations between pain characteristics and depression with opioid misuse risk were computed after stratifying by pain location. To assess whether there were any differential effects of depression or opioid misuse on other variables based on pain location strata, the interaction term in an ANOVA (for continuous variables) and the Breslow-Day test (categorical variables) were conducted. Last, a hierarchical logistic regression was conducted to calculate odds ratios and 95% confidence intervals by first adding pain characteristics and then adding depression, pain location, and the interaction of pain location and depression in successive blocks. All tests were conducted at *p* < 0.05.

## 3. Results

Table 1 displays the characteristics of the 122 pain patients included in our study sample. On average, patients were 49.6 (± 12.6) years of age, 56.6% female and nearly 80% white. One-fourth were positive for opioid misuse risk and 35.2% met the criteria for depression. The average total pain score was 6.3 (± 1.7) and average pain interference was 6.7 (± 2.4). Comparisons between CPOL and CLBP indicated that those with CLBP had lower average pain severity, lower average least pain and lower average pain than those with CPOL.

Table 2 shows the association between opioid misuse and pain characteristics by depression, stratified by pain location. Among CPOL patients, 66.7% of depressed patients were positive for opioid misuse compared to 7.5% of non-depressed CPOL patients (*p* < 0.001). Among CLBP patients, depression was not related to positive opioid misuse (*p* = 0.19). A Breslow-Day test showed that depression and opioid misuse were more strongly associated among CPOL than among CLBP patients (*p* = 0.019). Average COMM score was significantly greater among depressed compared to non-depressed CPOL (*p* < 0.0001) and CLBP (*p* < 0.01) patients; however the difference between depressed and non-depressed was larger (interaction *p*-value = 0.016) among CPOL (difference = 13.0) patients than CLBP patients (difference = 5.8). Among CPOL, but not among CLBP patients, average pain severity was significantly higher among depressed *vs.* non-depressed patients. Pain interference was significantly greater among depressed *vs.* non-depressed patients in both CPOL and CLBP patients. The relationship of BPI measures and depression were similar across pain location strata.

Table 3 shows the association between PHQ-9 score and pain characteristics by opioid misuse, stratified by pain location. The average PHQ-9 scores were significantly higher among COMM positive CPOL patients compared to COMM negative CPOL patients. The association between COMM status and PHQ-9 score was not significant in the CLBP group. There were marginally significant between strata differences in the relationship of opioid misuse and PHQ-9 score (interaction *p*-value = 0.050). Both pain severity and pain interference were positively related to COMM positive status among CPOL patients. Conversely, only pain interference was higher among COMM positive CLBP patients compared to COMM negative CLBP patients. There were no between strata differences in the relationship of opioid misuse and pain characteristics.

Table 4 shows results of a hierarchical logistic regression. In model 1, pain severity and interference explain 32.0% of the variance in the likelihood of opioid misuse risk, with each unit increase in pain interference having over twice the likelihood of opioid misuse risk (OR = 2.25; 95% CI: 1.45–3.48); however, pain severity was unrelated to opioid misuse risk. Adding depression explained an additional 5.2% (*p* = 0.019) of the variance in opioid misuse risk and increased the odds of opioid misuse risk by over 3-fold (OR = 3.32; 95% CI: 1.20–9.16). Pain location did not significantly explain any additional variance in opioid misuse risk; however, adding an interaction term of pain location and depression in a final model explained an additional 4.5% of the variance (*p* = 0.025). The significant interaction term showed that the odds ratio for the relationship of depression and opioid misuse risk increased by over a factor of 10 comparing CPOL to CLBP (depression OR: 10.57 *vs.* 1.04, respectively).

## 4. Discussion

In patients seeking treatment at a family medicine pain clinic, we observed evidence that the association between depression and opioid misuse differed in patients with CLBP compared to CPOL. Results of bivariate analysis stratified on pain location revealed that depression was more strongly associated with opioid misuse among CPOL patients than in patients with CLBP. This observation is not explained by pain interference which showed similar significant associations with depression in both CPOL and CLBP. Exploratory hierarchical logistic regression found that depression increases the odds of opioid misuse after adjusting for pain location and pain characteristics (severity and interference) and that this relationship is over 10 times stronger in CPOL than in CLBP. While pain severity was not significantly associated with opioid misuse risk in adjusted analysis, results suggest that pain interference independently contributes to opioid misuse risk after accounting for depression.

Our data expand on the existing literature on the prevalence of depression among different pain types [4,5,6,7,8,9] and the prevalence of opioid misuse across different pain types [3] by showing prevalence estimates of both opioid misuse and depression in groups stratified by pain location. Additional studies are warranted to determine if the frequent depression—opioid misuse comorbidity varies in CLBP compared to other specific types of pain such as arthritis, migraine, fibromyalgia, *etc.*

Because neuroticism is thought to be more common in CLBP [5] we expected CLBP patients to have more depression and subsequently more opioid misuse. However our results indicate the prevalence of depression does not differ by pain location. We did not expect to find a stronger association of depression and opioid misuse among CPOL patients. Though speculative, these results may be due to evidence from Morasco *et al*. [3] that neck and joint pain patients are more likely to have a history of substance use disorder compared to CLBP patients (94.4% *vs.* 83.3%).

One potential explanation for the stronger association between depression and opioid misuse in CPOL patients could be the significantly higher BPI score, higher average least pain and higher average pain level among CPOL *vs.* CLBP. In addition BPI scores were significantly greater in the depressed CPOL compared to non-depressed CPOL patients but did not differ by depression status in the CLBP patients. BPI scores were also higher in COMM positive *vs.* COMM negative patients in the CPOL patients but not CLBP patients. Greater pain severity in CPOL may lead to both depression and opioid misuse in patients with CLBP. Although pain severity was not significant when modeled with pain interference in regression analysis, this does not preclude the possibility that pain severity leads to greater pain interference which then increases depression and opioid misuse. Longitudinal data collection is needed to confirm this possibility.

There are several biological mechanisms proposed for the association between pain and depression. Common vulnerability due to past psychopathology or trauma leads to chronic pain via changes in catecholamines, substance P and cytokine activity and less responsive opioid receptors that may perpetuate or worsen depression [14]. Another biological underpinning for the pain-depression relationship is inflammatory processes and oxidative stress [15]. Studies of memantine, an NMDA receptor antagonist improves both pain and depression via reduction in glutamate activity and thereby reducing inflammatory factors related to pain and depression [16].

Limitations include small sample size which limits the precision of our conclusions. We did not have enough subjects to compare CLBP to specific types of CPOL such as arthritis or fibromyalgia; however, sensitivity analysis removing fibromyalgia which is often comorbid with depression, did not change our conclusions. We also lacked sample size to conduct stratified multivariable logistic regression models. The data did not contain type of opioid or co-medication which may bias results if CLBP or CPOL systematically differed in morphine equivalent dose because higher daily opioid morphine equivalent dose is associated with depression [17,18] and opioid misuse [19]. Comorbid conditions such as specific co-occurring pain conditions and anxiety disorders, were not available to improve control of confounding. The results may not apply to other geographic locations or to pain patients seeking care in other settings.

## 5. Conclusions

We found depression was more strongly associated with opioid misuse in patients with CPOL compared to patients with CLBP. The well-established association between depression and opioid misuse may be less prominent in CLBP than in other pain types. Further research with larger samples should compare this association in CLBP, arthritis, fibromyalgia and neuralgia. Refining which pain patients are most likely to develop comorbid depression and opioid misuse can inform clinical care and target limited resources to patients at greatest risk for this comorbidity.

## Figures and Tables

**Table 1:** Characteristics of family medicine pain clinic patients, n = 122.

Variables, Mean (sd)	Total (n = 122)	CPOL (n = 61) ^1^	CLBP (n = 61) ^1^	*p*-value
Age	49.6 (± 2.6)	50.4 (± 11.5)	48.8 (± 13.0)	0.485
Gender, n(%)				0.100
Female	69 (56.6%)	39 (63.9%)	30 (49.2%)	
Male	53 (43.4%)	22 (36.1%)	31 (50.8%)	
Race, n(%)				0.501
White	97 (79.5%)	47 (77.0%)	50 (82.0%)	
Non-white	25 (20.5%)	14 (23.0%)	11 (18.0%)	
Positive opioid misuse (COMM ^2^ > 9), n(%)	30 (24.6%)	17 (27.9%)	13 (21.3%)	0.400
COMM score ^2^	9.2 (± 9.1)	9.5 (± 9.8)	8.9 (± 8.5)	0.639
Depression (PHQ-9 ^3^ > 14), n(%)	43 (35.2%)	21 (34.4%)	22 (36.1%)	0.850
PHQ-9 ^3^ score	11.0 (± 6.7)	11.0 (± 6.8)	11.0 (± 6.7)	0.989
BPI ^4^ mean, (sd)				
Total Average pain index	6.3 (± 1.7)	6.6 (± 1.8)	5.9 (± 1.7)	0.035
Worst pain level	7.7 (± 1.7)	7.9 (± 1.7)	7.4 (± 1.6)	0.112
Least pain level	5.0 (± 2.2)	5.4 (± 2.2)	4.5 (± 2.1)	0.029
Pain level on average	6.3 (± 1.8)	6.7 (± 1.8)	6.0 (± 1.7)	0.039
Pain level right now	6.2 (± 2.3)	6.5 (± 2.3)	5.9 (± 2.2)	0.115
Total Average pain interference	6.7 (± 2.4)	6.7 (± 2.6)	6.7 (± 2.2)	0.900
General activity	7.2 (± 2.3)	7.2 (± 2.5)	7.2 (± 2.2)	0.957
Mood	6.5 (± 2.8)	6.4 (± 2.9)	6.6 (± 2.7)	0.713
Walking ability	6.7 (± 2.8)	6.7 (± 2.9)	6.7 (± 2.6)	0.987
Normal work	7.5 (± 2.6)	7.4 (± 2.7)	7.5 (± 2.5)	0.755
Relations with others	5.2 (± 3.4)	5.3 (± 3.6)	5.1 (± 3.3)	0.762
Sleep	6.8 (± 2.8)	6.8 (± 2.8)	6.8 (± 2.8)	0.987
Enjoyment of life	7.0 (± 2.9)	6.9 (± 3.2)	7.1 (± 2.7)	0.658

Note: *p*-value is for chi-square tests for categorical variables and independent samples *t*-tests for continuous variables. ^1^ CPOL: Chronic pain of other location, CLBP: Chronic low back pain; ^2^ COMM= Chronic Opioid Misuse Measure (0–68); ^3^ PHQ-9 = Depression scale (0–27); ^4^ BPI = Brief Pain Inventory (Average Severity and interference: 0–10, Individual BPI items: 0–10).

**Table 2:** Distribution of patient opioid misuse risk and pain characteristics by depression, stratified by pain location (n = 122).

Variables, Mean(sd)	CPOL ^1^ (n = 61)			CLBP ^1^ (n = 61)			Interaction *p*-value ^4^
	Not depressed (n = 40)	Depressed (n = 21)	*p*-value	Not depressed (n = 39)	Depressed (n = 22)	*p*-value	
Positive opioid misuse (COMM ^2^ > 9), n(%)	3 (7.5%)	14 (66.7%)	<0.001	6 (15.4%)	7 (31.8%)	0.19	0.019
COMM ^2^	5.1 (± 5.1)	18.1 (± 10.9)	<0.001	6.8 (± 7.5)	12.6 (± 9.1)	0.01	0.016
BPI ^3^							
Total average pain index	6.1 (± 1.8)	7.6 (± 1.2)	<0.001	5.8 (± 1.7)	6.3 (± 1.6)	0.25	0.143
Total average pain interference	5.7 (± 2.6)	8.5 (± 0.9)	<0.001	6.1 (± 2.1)	7.8 (± 1.9)	0.004	0.151

Note: *p*-value is for chi-square tests for categorical variables and independent samples *t*-tests for continuous variables. ^1^ CPOL: Chronic Pain of Other Location, CLBP: Chronic Low Back Pain; ^2^ COMM = Current Opioid Misuse Measure (0–68); ^3^ BPI = Brief Pain Inventory (Average Severity and interference: 0–10); ^4^ *p*-value for interaction term of pain location x depression, ANOVA for continuous variables and Breslow-Day test for dichotomous variables.

**Table 3:** Distribution of depression score and pain characteristics by opioid misuse, stratified by pain location (n = 122).

Variables, mean (sd)	CPOL ^1^ (n = 61)			CLBP ^1^ (n = 61)			Interaction *p*-value ^5^
	COMM ^2^ negative (n = 44)	COMM ^2^ positive (n = 17)	*p*-value *	COMM ^2^ negative (n = 48)	COMM ^2^ positive (n = 13)	*p*-value *	
PHQ-9 ^3^	8.6 (± 5.6)	17.4 (± 5.2)	<0.001	10.3 (± 6.8)	13.9 (± 5.7)	0.08	0.050
BPI ^4^							
Total Average pain index	6.3 (± 1.8)	7.5 (± 1.3)	0.017	5.8 (± 1.7)	6.6 (± 1.6)	0.101	0.638
Total Average pain interference	5.9 (± 2.6)	8.6 (± 1.0)	<0.001	6.3 (± 2.2)	8.2 (± 1.2)	0.005	0.384

Note: *p*-value is for chi-square tests for categorical variables and independent samples *t*-tests for continuous variables. ^1^ CPOL: Chronic Pain of Other Location, CLBP: Chronic Low Back Pain; ^2^ COMM = Current Opioid Misuse Measure (0–68); ^3^ PHQ-9 = Depression scale (0–27); ^4^ BPI = Brief Pain Inventory (Average Severity and interference: 0–10); ^5^ *p*-value for interaction term of pain location * COMM Opioid Misuse, ANOVA for continuous variables and Breslow-Day test for dichotomous variables.

**Table 4:** Logistic regression models for opioid misuse risk (COMM ^1^) (n = 122).

Predictor Variables	Model 1. Pain severity and interference	Model 2. Add depression	Model 3. Add pain location	Model 4.Add depression * pain location
Variables	OR (95% CI)	OR (95% CI)	OR (95% CI)	OR (95% CI)
Total average pain severity ^2^	0.88 (0.60–1.29)	0.92 (0.61–1.37)	0.87 (0.58–1.31)	0.81 (0.52–1.26)
Total average pain interference ^2^	2.25 (1.45–3.48)	1.91 (1.21–3.01)	1.95 (1.23–3.09)	2.09 (1.26–3.47)
Depression (Yes *vs.* No)		3.32 (1.20–9.16)	3.32 (1.19–9.23)	
Pain Location ^3^				
CPOL			1.00	-
CLBP			0.64 (0.23–1.77)	-
Depression * Pain Location ^4^				
CPOL: Depression (Yes *vs.* No)				10.57 (2.21–50.49)
CLBP: Depression (Yes *vs.* No)				1.04 (0.25–4.41)
Chi-square change (df, *p*-value)	29.58 (2, <0.0001)	5.48 (1, 0.019)	0.74 (1, 0.390)	5.00 (1, 0.025)
Nagelkerke R-square	0.320	0.372	0.378	0.423

Note: OR = odds ratio; CI = confidence interval; ^1^ COMM = Chronic opioid Misuse Measure; ^2^ Brief Pain Inventory (Average Severity and interference: 0–10), odds ratio represents the change in odds of opioid misuse risk given one unit increase in average pain severity or interference; ^3^ CPOL: Chronic pain other location, CLBP: Chronic low back pain; ^4^ Wald Chi-square *p* = 0.031.

## Abbreviations

The following abbreviations are used in this manuscript:

CLBP	Chronic low back pain
CPOL	Chronic pain of other location
COMM	Current Opioid Misuse Measure
PHQ-9	Patient Health Questionnaire-9
BPI	Brief Pain Inventory
